# Factors Influencing Medical Interns’ Choice of Hospital for Training, Saudi Arabia: A Cross-Sectional Study

**DOI:** 10.7759/cureus.54187

**Published:** 2024-02-14

**Authors:** Farooq U Pasha, Roaa Aljumaa, Ghada Almasri, Miral Atout, Joudi Baladi

**Affiliations:** 1 Emergency Department, King Faisal Specialist Hospital and Research Centre, Riyadh, SAU; 2 Medicine, Alfaisal University College of Medicine, Riyadh, SAU

**Keywords:** undergraduate, medical education, saudi arabia, medical internship experience, internship

## Abstract

Background

High-quality, adequately resourced, and strategically oriented medical internship training plays a pivotal role in the continual growth of junior doctors. Evaluating the perspectives of final-year medical students regarding their internship year and their preferred placement is of paramount importance. This study aims to furnish internship programs with valuable insights into interns' perceptions and attitudes toward an optimal internship experience. By doing so, we aim to empower internship programs with the necessary knowledge to enhance their offerings, ensuring they are better tailored to the needs and preparation of future doctors.

Objective

The objective of this study was to elucidate the primary factors that influence the preferences of medical interns in selecting a hospital for their internship training.

Methods

In the academic year spanning September 2023 to 2024, a comprehensive cross-sectional study was conducted at three esteemed medical universities in Riyadh, Saudi Arabia. A meticulously crafted questionnaire, consisting of twenty questions was disseminated via various social media platforms, targeting all final-year medical students of the selected medical schools. Overall 241 students actively participated in the survey, their valuable contributions enriching the overall quality and depth of the research findings.

Results

Among the 241 respondents, 67.22% were male, 83.40% identified as Saudi, and 43.57% hailed from King Saud bin Abdulaziz University for Health Sciences (KSAU-HS). A predominant 94.61% of participants fell within the age group of 22-25 years. Notably, the top four influential factors influencing participants’ choice of internship training programs were encouraging consultants (93.80%), the hospital working environment (91.29%), an internship program with effective teamwork (88.38%), and a subspecialty residency program (85.48%). Furthermore, our findings underscored that access to electronic health record (EHR) systems, orientation prior to clinical rotations, and seniors' experiences significantly impact students' decisions when selecting a hospital for rotations. It was also observed that considerations such as the number of on-calls and working hours, salary, location, and extracurricular activities wield a substantial influence over their choices, as indicated by the majority of the surveyed students.

Conclusion

Given that internship training is one of the most critical stages of medical education, it is recommended to take into account the elements that students believe can influence their hospital choice for conducting their internship. We hope that by acknowledging these aspects, potential approaches to improve and advance hospital training can be formulated in order to provide interns with effective training, a reasonable workload, and a conducive and supportive environment to work in.

## Introduction

It is a well-recognized and established fact that a medical graduate's internship period, a time when a medical student transforms into a fully licensed doctor, is a challenging yet absolutely essential transitional phase [1]. According to the National Board of Health and Welfare in Sweden, a medical internship should serve the objective of exposing a recently graduated doctor to clinical work by allowing him or her to acquire knowledge and experience based on what was learned in medical school [2]. Therefore, if the training during the internship is not well-resourced and appropriate, interns will be more stressed and will not get the required experience they need in their specialties [3,4]. There are numerous factors that impact how effective training is, and interns' opinions are rarely taken into consideration [5]. To ensure interns' satisfaction and fruitful medical intern training programs, several factors should be taken into consideration: a conducive environment, sufficient opportunities for experiential learning, a reasonably balanced workload, good supervision, and an effective support system [6].

Satisfied medical interns are more likely to acquire crucial skills and gain confidence in their medical practice, which will improve their ability to care for patients in the future. A study conducted in four medical schools in Saudi Arabia determined some factors influencing medical interns’ satisfaction with their programs, such as training site services, orientation, supervision, relationships with superiors, and hospital activities [7]. Moreover, a descriptive-analytical study carried out among interns at Guilan University of Medical Sciences revealed that they were dissatisfied with the clinical training objectives, evaluation methods, equipment and facilities, and the student's clinical skills level [8]. This shows that such factors determine where interns decide to do their rotations. The aim of this study is to shed light on the principal factors that influence where medical graduates want to undergo their internship training. This analysis may help future Saudi hospitals focus on major areas for improvement in order to provide the best training program for interns.

This article was initially showcased as a poster during the 14th Annual Student Poster Competition at Alfaisal University on March 2, 2023, and later at the European Emergency Medicine Congress (EUSEM) 2023 held in Barcelona from September 16 to 20.

## Materials and methods

A quantitative study was conducted at three medical schools in Riyadh, Saudi Arabia, between September 2023 and 2024. All final-year medical students of the academic year 2024 at Alfaisal University, King Saud bin Abdulaziz University for Health Sciences (KSAU-HS), and Imam Muhammad Ibn Saud Islamic University were eligible for inclusion in this study. These three universities were chosen due to easier access for medical graduates. Interns, undergraduate medical students except final-year medical students, and medical graduates outside Riyadh, Saudi Arabia were excluded from the study. An IRB approval was obtained from King Faisal Specialist Hospital and Research Centre (IRB-2231252).

After meticulously reviewing the literature of articles published from 2008 to 2023 and enquiring the opinion of several medical educational specialists, a self-structured questionnaire incorporating 20 items was developed to assess the principle factors that influence where medical graduates favor performing their internship training. The questionnaire was reviewed by the research team and then sent to a small sample for validation.

A short briefing regarding the main aim of the research was given at the beginning of the survey. The first section covered demographics such as age, gender, nationality, and the university the students attended. The second section included questions that allowed us to explore our research objectives in depth. An open-ended question was added at the end to give the participants the opportunity to share any other factors that may influence their decision. The questionnaire was then disseminated via social media platforms such as WhatsApp.

A total of 200 medical graduates were considered at each university. Therefore, a sample size of 235 was obtained after applying a 95% confidence interval and a 5% margin of error to the full census of the final-year students from the three universities (n=600). Participants were requested to indicate their level of agreement with each item on a five-point Likert scale. The Likert scale was used, 5=strongly agree, 4=agree, 3=neutral, 2=disagree, and 1=strongly disagree. All students consented before participating in the study and had the choice to participate or withdraw at any time from the study. Their participation was anonymous, with no personal information required, and the confidentiality of respondents’ data in the research was promised and maintained. Only research team members had access to the collected data.

Statistical analysis

A descriptive statistical analysis of the results was performed using Statistical Package for the Social Sciences (SPSS Inc., Chicago, IL), version 25, as frequency tables, percentages, and graphs. A p-value of <0.05 was considered statistically significant in all analyses. 

## Results

Table 1 illustrates the baseline characteristics of the study participants. Out of the 241 responses, 162 (67.22%) were males, 201 (83.40%) were Saudi, and the majority (43.57%) of the respondents were from KSAU-HS. Responses from medical graduates outside KSAU-HS, Imam Muhammad Ibn Saud Islamic University, and Alfaisal University were eliminated from the study. Of the 241 participants, the majority (94.61%) were from the age group of 22-25 years old, while only 13 (5.39%) fell under the age group of 26-30 years old.

**Table 1:** Baseline characteristics of the study participants KSAU-HS, King Saud bin Abdulaziz University for Health Sciences

Demographic characteristics	N (%)	
Gender		
Male	162 (67.22%)	
Female	79 (32.78%)	
Nationality		
Saudi	201 (83.40%)	
Non-Saudi	40 (16.60%)	
Place of study		
KSAU-HS	105 (43.57%)	
Imam Muhammad Ibn Saud Islamic University	84 (34.85%)	
Alfaisal University	52 (21.58%)	
Age		
22-25		228 (94.61%)
26-30		13 (5.39%)

Table 2 demonstrates the top four influencing factors, which include encouraging consultants, hospital working environment, internship program with effective teamwork, and subspecialty residency program. A notable 93.78% of the students would choose hospitals that have encouraging consultants who provide students letters of recommendation as per their efforts, underscoring the importance of mentorship and recognition in their professional development (p < 0.01). Similarly, the hospital atmosphere played a crucial role in their decision-making, with 91.29% favoring friendly and motivating staff, a safe and healthy environment, and a good reputation (p < 0.01). A culture of teamwork was preferred by 88.38% (p < 0.01). The availability of interested sub-specialties influenced the choices of 85.48% of the participants (p < 0.01).

**Table 2:** Top four factors influencing internship training

Factors	Agree	Neutral	Disagree
Encouraging consultants, n (%)	226 (93.78%)	12 (4.98%)	3 (1.24%)
Hospital working environment, n (%)	220 (91.29%)	13 (5.39%)	8 (3.32%)
Effective teamwork, n (%)	213 (88.38%)	21 (8.71%)	7 (2.91%)
Subspecialty residency program, n (%)	206 (85.48%)	16 (6.64%)	19 (7.88%)

**Figure 1:**
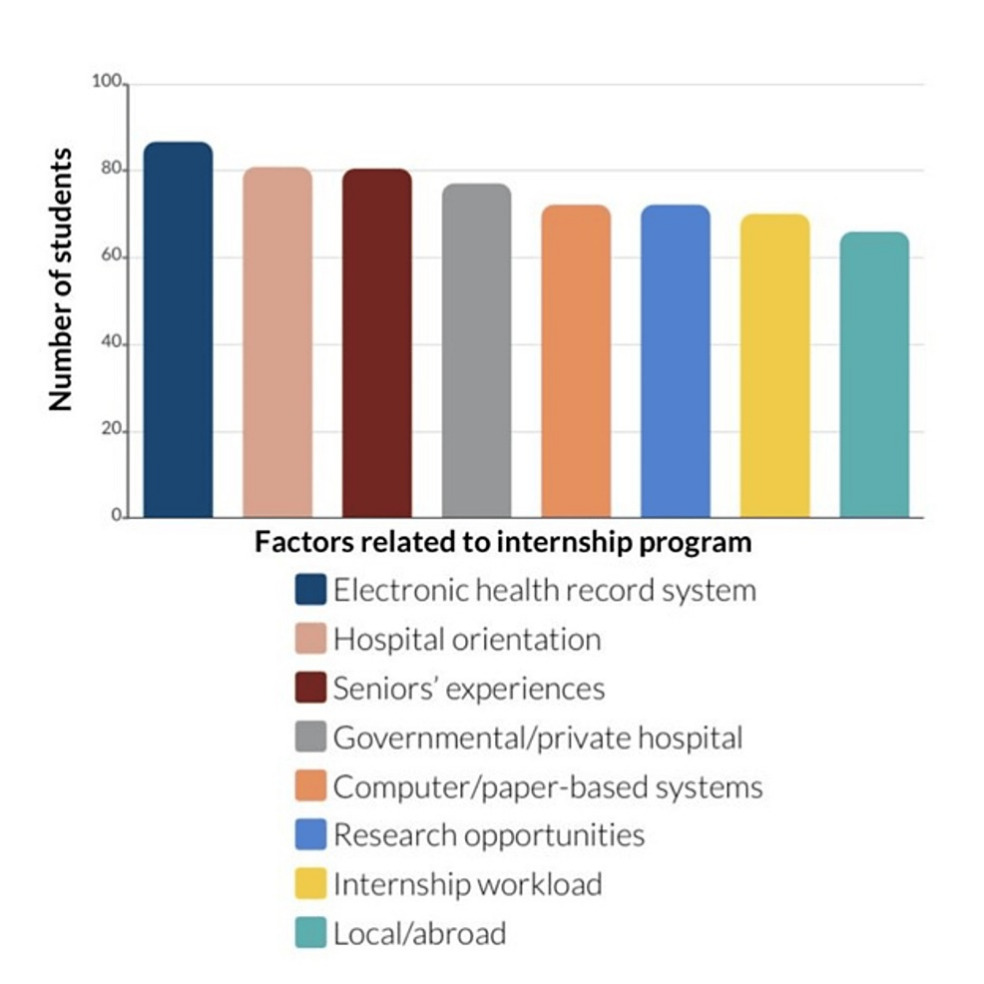
Factors related to the internship program

Figure 1 represents the factors related to the internship program. A significant 86.72% of the respondents would rather do their internship in hospitals that provide interns with access to electronic health record (EHR) systems (p < 0.01). The survey results indicated a strong preference among medical graduates for hospitals that provide orientation prior to the start of their internship, as 80.91% of respondents agreed on its importance (p < 0.01). Regarding the experiences shared by senior colleagues in healthcare, 80.50% of the respondents showed the major impact it has on medical graduates who want to pursue their internship training (p < 0.01). Additionally, 77.18% showed a preference for governmental hospitals, attributed to the diversity of case presentations (p < 0.01). Furthermore, 72.20% of the students agreed that it is of value to them whether the hospital has a computer system or a paper-based system, and an equal percentage would opt for hospitals that offer research opportunities (p < 0.01). Upon further analysis, it was found that 70.12% of the participants agreed that the responsibilities, or daily workload, given by the hospital can influence someone’s choice (p < 0.01). Finally, 65.98% would like to perform their internship training locally rather than abroad (p < 0.01).

**Figure 2:**
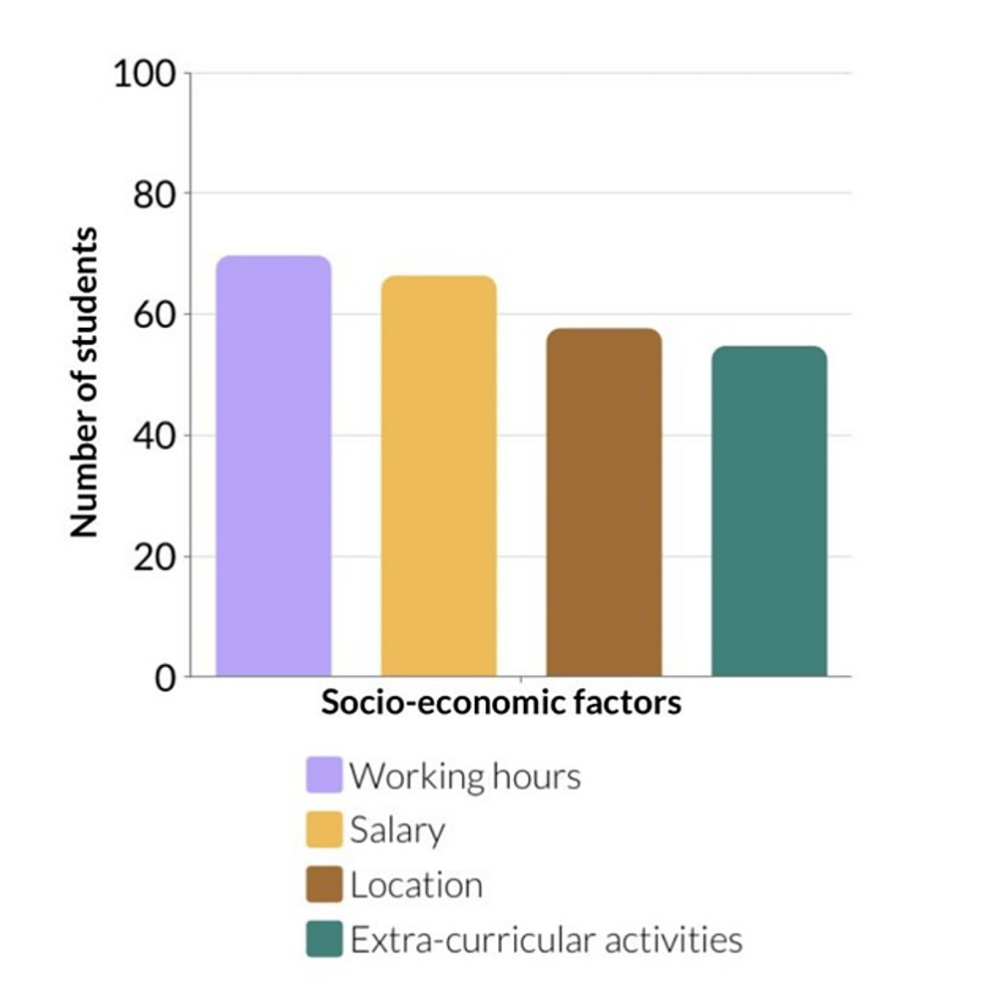
Socio-economic factors influencing the choice of internship

Figure 2 displays the socio-economic factors that influence the choice of internship, which are working hours, salary, location, and extracurricular activities. The survey highlighted the importance of manageable on-call and working hours, as it was a selection criterion for 69.71% of the respondents (p < 0.01). Furthermore, getting paid by the hospital was a concern for 66.39% of the graduates (p < 0.01). Practical concerns such as the hospital's location relative to their home influenced 57.68% (p < 0.05). 54.77 % of the 241 respondents would consider hospitals that provide extracurricular activities (awareness campaigns and conferences) (p < 0.05).

## Discussion

​​​Unlike the majority of studies on the subject, this research did not assess how satisfied interns were with their training during their internship. Instead, our purpose was to evaluate the variables affecting where medical school graduates in Saudi Arabia want to complete their internship training. To the best of our knowledge, no research has been conducted on this aspect of internships. Our major findings revealed that encouraging consultants, a hospital working environment, internship programs with strong teamwork, and subspecialty residency programs are the top four influencing factors that have an impact on medical graduates' decision to pursue internship training. On the other hand, the least three determining criteria were salary income, distance from home, and extracurricular activities.

As considerable emphasis is given to the letter of recommendation within the context of the residency application, a significant proportion of students (93.78%) prefer hospitals with supportive consultants who acknowledge their efforts by providing letters of recommendation [9]. The positive reinforcement also facilitates transparent communication, leading interns to actively seek guidance, ask questions, and engage in their learning journey when they sense encouragement from consultants. From our perspective, this communication is crucial for interns to gain practical experience. Several publications describing the working environment in hospitals were highlighted in our literature study, demonstrating the significance of this factor. As evident by our findings, 91.29% of the students think that an internship's quality and efficacy are directly influenced by the working environment. In a study conducted in 2020 evaluating interns' perspectives on their internship programs in Saudi Arabia, the findings indicated that there is a need to enhance the hospital environment for interns to establish effective interactions with all hospital staff, patients, and attendants [7]. Furthermore, a 2008 study exploring the perceptions of South African interns working in regional hospitals showed that the trainers did not always have a positive attitude toward the atmosphere in a hospital. It was crucial for the intern to have a good working relationship with the senior doctor when learning procedures. As a result, the intern had to exhibit a level of passion to encourage his coworkers [10]. Another study showed that a learning environment, a positive social atmosphere, good supervision, management support, the interns' positive attitudes, and teamwork all contribute to the effective training of medical interns [11]. Accordingly, a supportive hospital environment is essential for our participants, as it positively impacts interns' well-being, job satisfaction, and success in their medical careers.

The majority of respondents (88.38%) favor medical facilities that value teamwork, which is supported by a South African study in 2012 highlighting the positive influence of teamwork on intern training [11]. To work as a team, healthcare professionals need to get along well with one another. Although there was no official training on such life skills, having good interpersonal relationships decreases conflicts at work, and internship training offers the opportunity to build communication and teamwork skills, which are equally vital in overcoming personal challenges [11]. Overall, teamwork in hospitals can lead to better collaboration, communication, and patient care, benefiting both interns and the healthcare team as a whole.

During a traditional medical internship, interns undergo rotations in departments like internal medicine, surgery, pediatrics, and more. However, some internships provide subspecialty rotations in areas like cardiology, neurology, gastroenterology, and dermatology. As a result, the availability of the specialty they are interested in has an impact on where medical graduates choose to continue their internship training, according to 85.48% of them. The presence of subspeciality rotations enhances interns' learning by exposing them to various medical conditions and treatments, facilitating the exploration of different specialties, and making informed decisions about their future career paths.

A significant portion of our participants (86.72%) choose to do their internships in medical facilities that give interns access to EMR systems. In this respect, several studies conducted in Saudi Arabia showed that healthcare professionals' opinions about EMRs are positive, and they agree that the EMR system boosts productivity and increases healthcare workers’ capacity to accomplish their tasks significantly faster than before [12-14]. Moreover, the EMR system gives them the precise data they want; the templates are appropriate for their specialty; and the system expands their capacity to add significant content [13]. As a result, many expressed a preference for EMR systems due to the numerous benefits they provide in terms of data accessibility, efficiency, and improved patient care.

At the start of the internship training, orientation is advised to lay out the educational goals so that the students are familiar with them. Orientation programs before the clinical rotation began were preferred by 80.91% of those surveyed. This result was consistent with a 2017 study stating that pre-internship training programs significantly influence how prepared and confident interns feel about their knowledge and abilities in a range of training disciplines [15]. Furthermore, 80.50% of students believe that following the advice of their senior colleagues influences where they choose to complete their internship. A Saudi Arabian study indicated that most of the interns had some idea about what to expect from an internship, as the source of this knowledge was mostly seniors [5]. The literature also revealed that many newly qualified doctors in Saudi Arabia considered receiving inadequate career advice to be a significant barrier to making a decision about their future [16]. Consequently, it is crucial for recent medical graduates to have input from their more experienced peers since it will enable them to choose the most suitable hospitals for their internship.

Due to the variety of medical cases, 77.18% of students choose to receive their training at governmental hospitals, as they offer many learning opportunities for medical interns. Because of their ability to care for a large number of patients, particularly those with complex medical conditions, interns are exposed to a wide range of cases. However, it is crucial to highlight that these opinions might differ across medical interns since individual circumstances and viewpoints have a big impact on how they see public hospitals.

The use of paper-based systems in hospitals as opposed to computers was another factor we thought of evaluating among medical graduates. According to our research, 72.20% of them believe that it does matter whether the hospital uses a computer system or a paper-based one for its record-keeping. A 2014 study comparing paper-based systems with computerized ones for sepsis management revealed that the latter is linked to a lower hospital mortality rate and early sepsis detection [17]. Although some interns may still have preferences for either computer or paper-based systems, the healthcare industry is increasingly adopting computer-based systems primarily because of their effectiveness, convenient data access, and improved patient well-being. This suggests that the majority of interns are likely to prefer computer systems.

Based on the responses received, 72.20% of the students said they would choose hospitals that allow patients to participate in research initiatives. In support of previous research in Pakistan, 32% of final-year students and 20% of interns are interested in publishing, demonstrating the great interest of undergraduates and recent graduates in research [18]. Research experience during medical training can enhance a student's resume and open doors to prestigious residency programs, fellowships, and academic positions in the future. In addition, many students are eager to contribute to the advancement of medical knowledge.

The daily workload the hospital provides can have an impact on participants’ choices, according to 70.12% of them. As in most similar studies, a significant relationship was found between workload and interns’ satisfaction. A study conducted in Saudi Arabia showed that some interns felt the tasks they had been given did not provide them enough time to study for their entry, licensing, or postgraduate exams [7]. Hospital workload was one of the key factors discouraging interns from remaining at a hospital, as highlighted by a study analyzing factors influencing perceptual experiences in South Africa [6]. As students require time and energy to prepare for their entrance tests in order to be admitted into residency programs, interns carefully consider the work hours and burden during their internship period.

When we examined whether medical graduates preferred their internship training to be conducted locally or abroad, the findings revealed that 65.98% of the participants chose to complete their internship locally. In contrast to a South African study, the majority of interns expressed a desire to work abroad, while only a small percentage preferred staying at their current institution [6]. In our opinion, the contradiction discovered in our study can be attributed to differences in educational environments and the countries’ economic status.

In accordance with our results, the experience of an internship can be considerably impacted by four socio-economic factors: working hours, salary, location, and extracurricular activities. Many students (69.71%) concur that working hours affect their decision-making, and 66.4% of medical graduates prefer to be compensated by the hospital. In a Polish study, physicians reported the lowest levels of satisfaction with regard to salary, work-life balance, and the ability to maintain worthwhile extracurricular activities. Doctors' satisfaction was inversely correlated with the number of hours they worked each week [19]. A study conducted in 2014 to investigate the prevalence of stress among medical graduates during their internship training found that interns working longer hours tended to experience higher levels of stress in Saudi Arabian hospitals [20]. The hospital's location is another important consideration in the participants’ decision; 57.68% of medical students favor hospitals that are close to their homes and are conveniently accessible. The findings contradict the claims in an Australian survey, which found that only 13% of interns and residents considered traveling distance as a top factor influencing their choice of residency location [21]. However, the intern’s perception of the hospital’s location in relation to their homes is influenced by the ease or difficulty of their commute. When the hospital is situated near their residence, interns tend to have a favorable attitude due to the time saved and reduced stress associated with shorter distances. Extracurricular activities such as conferences and awareness campaigns might determine where students receive their training. 54.77% of the respondents in the current research choose hospitals that offer extracurricular activities. Hospital activities were another important aspect determining overall satisfaction with their internship training, according to Saudi Arabian findings [7].

While a key strength of this study is that it is the first that focuses on medical graduates’ views on internship training, there are nonetheless some limitations that should be addressed to facilitate a proper understanding of the results. First, it is a cross-sectional study, so there is a chance that reporting biases may have arisen due to inaccurate responses or the respondents' interpretation of the questions or desire to express their emotions in a particular way. Second, it is restricted to the three Saudi medical schools that were chosen for the study; hence, additional research at other medical schools in Saudi Arabia is advised to produce more comprehensive findings.

According to a study, when compared to residents and graduate students, interns had the highest stress scores, both overall and for each individual stress scale item [22]. Therefore, our findings may aid Saudi Arabian authorities in improving the internship training programs to ensure the overall satisfaction of medical interns, in addition to enhancing their academic performance and clinical training.

## Conclusions

Given that internship training is one of the most critical stages of medical education, it is recommended to take into account the elements that students believe could influence their hospital choice when choosing where to conduct their internship. Our key findings showed that encouraging consultants, hospital working environments, subspecialty residency programs, and internship programs with excellent teamwork are the top influencing factors that affect medical graduates' decisions. We hope that by acknowledging these aspects, training hospitals can further improve their internship programs for greater intern satisfaction.

## Appendices

We are a group of medical students conducting a cross-sectional study at Alfaisal University. We would like to invite you to take part in our research study focusing on the factors influencing which hospital medical graduates prefer to do their internship training in Riyadh, Saudi Arabia.

As a valued member of our community, your input will make a huge difference in future internship programs. Completing the survey will only require three to five minutes of your time. Your responses are completely anonymous, and your participation is entirely voluntary, you may withdraw at any point.

Your participation is valued, and we would appreciate it if you complete the online survey below. Please note that the survey is open to all medical graduates at Alfaisal University, King Saud bin Abdulaziz University for Health Sciences (KSAU-HS), and Imam Muhammad Ibn Saud Islamic University.

Thank you in advance for your time and participation. If you have any questions, please do not hesitate to reach out to us.

Questionnaire questions:

Do you agree to participate in this study?

- Yes

- No

What is your gender?

- Male

- Female

What is your nationality?

- Saudi

- Non-Saudi

What is your age?

Where do you study?

- Alfaisal University

- King Saud bin Abdulaziz University for Health Sciences (KSAU-HS)

- Imam Muhammad Ibn Saud Islamic University

Are you a final-year medical student?

- Yes

- No

Please answer the following questions with Strongly Agree, Agree, Uncertain, Disagree, Strongly Disagree.

1. I prefer going to hospitals that provide orientation prior to the start of the clinical rotation.

2. The hospital’s atmosphere is of concern to me (friendly and motivating staff, safe and healthy environment, and good reputation).

3. Seniors’ experiences influence my decision on where to conduct my internship training.

4. My interest in a certain residency program influences where I choose to train.

5. Having to prepare for board exams during internship affects my decision on where to train.

6. I prefer training in governmental hospitals due to the diversity of case presentations.

7. I consider the hospital’s infrastructure when choosing the place of my residency program (protocols, rules, and arrangement of departments).

8. I would rather do my internship in hospitals that provide interns with access to electronic health record systems.

9. I prefer hospitals that foster a culture of teamwork.

10. I would choose hospitals that have encouraging consultants who provide students with letters of recommendation as per their efforts.

11. It is of value to me whether the hospital has a computer system or a paper-based system.

12. The location of the hospital from my home influences my decision.

13. The responsibilities or daily workload given by the hospital can influence my hospital choice (case presentations, journal club).

14. Getting paid by the hospital influences my decision.

15. I would consider hospitals that provide extracurricular activities (awareness campaigns, conferences).

16. Number of on-calls and working hours influences my selection.

17. The availability of the sub-specialty I’m interested in affects my choice.

18. I would choose hospitals that offer opportunities to be part of research projects.

19. I would like to perform my internship training locally rather than abroad.

20. As a non-Arab, the hospital’s dominant language affects my choices of training hospitals.

Short answer question: What are other factors that may influence your decision?
